# Chitosan-Folic Acid-Coated Quercetin-Loaded PLGA Nanoparticles for Hepatic Carcinoma Treatment

**DOI:** 10.3390/polym17070955

**Published:** 2025-03-31

**Authors:** Anil Kumar Sahdev, Chaitany Jayprakash Raorane, Mohammad Ajmal Ali, Khalid Mashay Al-Anazi, Ranjith Kumar Manoharan, Vinit Raj, Anita Singh

**Affiliations:** 1 Department of Pharmaceutical Sciences, Faculty of Technology, Sir J.C. Bose Technical Campus, Kumaun University, Nainital 263136, Uttarakhand, India; anilsahdev20@gmail.com; 2 School of Chemical Engineering, Yeungnam University, Gyeongsan 38541, Republic of Korea; drvinitraj@cau.ac.kr; 3 Department of Botany and Microbiology, College of Science, King Saud University, Riyadh 11451, Saudi Arabia; 4 Department of Zoology, College of Science, King Saud University, Riyadh 11451, Saudi Arabia; 5 Department of Civil Engineering, Yeungnam University, Gyeongsan 38541, Republic of Korea

**Keywords:** hepatic cellular carcinoma, quercetin, PLGA, chitosan, folic acid, nanoparticles

## Abstract

Hepatocellular carcinoma (HCC) causes the third highest mortality worldwide. Liver ablation, surgery, and embolization are conventional methods for treatment. However, these methods have limitations. To overcome these issues, nanomedicines have potential due to their high stability, high drug load capacity, and controlled release. Thus, we prepared quercetin-loaded polylactic-co-glycolic acid (PLGA) nanoparticles coated with folic acid-chitosan (QPCF-NPs) to improve drug delivery and targetability applications of quercetin for the treatment of HCC. We prepared QPCF-NPs by solvent evaporation and coated them with chitosan-folic acid (CS-FA). QPCF-NPs were examined using Fourier-Transform infrared (FTIR), scanning electron microscopy (SEM), transmission electron microscopy (TEM), and X-ray diffraction (XRD). In addition, the drug release rate and cytotoxicity were studied. Moreover, in vivo HCC studies such as histopathology and biochemical parameters were conducted. Subsequently, QPCF-NPs with a spherical shape and an average size of 200–290 nm have been demonstrated to have formed by FTIR, XRD, SEM, and TEM. Further, we observed sustained drug release from QPCF-NPs compared to quercetin. Cellular cytotoxicity showed significant inhibition in the HEPG2-cell line with QPCF-NPs treatment. Biochemical estimate and oxidative stress regulation were considerably more regulated in the treatment groups than the HCC group in a dose-dependent way after subcutaneous administration of QPCF-NPs. ELISA of interleukin and caspase-3 demonstrated the anticipated results in comparison to the carcinogen control group. Compared to earlier preparations, the QPCF-NPs generated demonstrated better drug targetability and potency for treating HCC.

## 1. Introduction

The third leading cause of cancer is hepatocellular carcinoma (HCC), a prevalent cause, and the sixth most common cancer type [1]. According to GLOBOCAN 2022, nearly 20 million new HCC cases were reported in 2022, with approximately 9.7 million deaths attributed to the disease [2]. Among all cancers, lung cancer topped the list as the primary cause of 31 cancer-related fatalities, which is thought to be around 1.8 million deaths (18.7%) [2]. HCC is the sixth most common kind of cancer and the third most common cause of death [3]. The World Health Organization reported in 2008 that 7.6 million people died from HCC, increasing the risk of death in developing nations [4]. In addition to gathering the latest data on the incidence of cancer in populations, the American Cancer Society predicts the numeral of innumerable types of the annual number of new cancer cases and deaths in the United States and results utilizing the National Center for Health Statistics’ (in 2021) mortality data and central malignance registries’ (in 2020) rate data [5]. According to estimates, the United States will have 2,001,140 additional new incidents of cancer and 611,720 deaths from cancer disease in 2024 [5].

The liver can develop several types of cancer [6]. Liver cancer can be primary, starting in the liver, or secondary, spreading to the liver from another part of the body. The most common type is HCC, beginning with hepatocytes; other less common types include intrahepatic cholangiocarcinoma and hepatoblastoma. Primary liver cancer primarily comprises HCC, accounting for 75–85% of cases, and intrahepatic cholangiocarcinoma, affecting 10–15% of cases. Chronic infection with HBV or HCV contributes to 21–55% of HCC cases globally [7]. This is the most difficult scenario for healthcare facilities because the demand for hepatic cancer treatment is increasing, which has negative economic effects. Chemotherapeutic drugs for cancer are typically toxic and cause drug resistance over time [8]. Drug concentration reductions in cells because of renal toxicity and target gene mutations are two examples of the factors that might contribute to drug resistance [9]. P53/mouse double minute-2 homolog (MDM2) inhibitors, angiogenesis inhibitors, hedgehog pathway blockers, tyrosine kinase inhibitors, mechanistic target of rapamycin (mTOR) inhibitors, histone deacetylase (HDAC) inhibitors [10], proteasome inhibitors, and poly (adenosine diphosphate-ribose) polymerase (PARP) inhibitors are among the promising inhibitor therapies for the treatment of solid cancers [11]. Despite the effectiveness of synthetic chemotherapeutic drugs for the treatment of HCC, chemoresistance poses significant challenges [12]. To improve on the available treatments against cancer, it would be interesting to evaluate the potential of naturally sourced drugs. Polymeric nanoparticles can be used in drug delivery systems as carriers [13,14].

Because of their excellent structural integrity, durability over time in storage, and capacity for controlled release, polymeric NPs have widespread application [15], and their capacity to deliver drugs to the therapeutic target at appropriate times and doses has garnered a lot of interest [16]. The benefits of using bipolymeric nanoparticles as modified drug carriers include the potential for controlled drug release, the enhancement of bioavailability and therapeutic index, and the protection of pharmaceuticals and other biologically active compounds from the environment [17]. The Food and Drug Administration has certified PLGA (polylactic-co-glycolic acid) as a drug material for drug delivery systems, and it is broadly utilized [18]. Multiple drug resistance in cancer cells mediated by P-glycoprotein (P-gp) can be decreased and drug permeability enhanced by PLGA, a great emulsifier for creating NPs [19].

*N*-acetyl glucosamine and β (1→4) combined glucosamine copolymerize to form the naturally occurring, non-toxic polymer known as chitosan [20]. In the food and bioengineering sectors, chitosan is widely utilized for regulated drug administration, enzyme immobilization, and encapsulating active food ingredients. Chitosan is valued for its biocompatibility, polycationic nature, bioactivity, biodegradability, and nontoxicity [21]. Folate receptors are overexpressed in various cancer cells. Folic acid (FA) is vital for DNA replication, with increased demand in promptly proliferating cells such as tumor cells [22]. This has led to the targeting of folate receptors as a key therapeutic strategy. FA is also used as a targeting agent in various formulations, either directly attached to therapeutics or via nanocarriers [21]. Chitosan nanoparticle (CS-NP)-delivered drugs selectively accumulate in tumoral, rather than normal, tissues due to the improved retention and permeability impact. Chitosan surface changes, in addition to their physicochemical characteristics, are essential for the cytotoxic profile and targeting of malignancies that exhibit aggressive growth and rapid division [23]. Chitosan oligomers have antitumor activity in vitro and in vivo [24], and chitosan-NPs showed in vitro cytotoxicity against the liver cancer cell line HepG2 [25].

Quercetin, an antioxidant flavonoid abundant in plant-based foods, plays a crucial role in combating free radical damage, inflammation, and aging [26]. Quercetin has weak water solubility, chemical stability, and absorption profile; however, it generally results in a relatively low bioavailability (<10%). The chemical structure, physicochemical characteristics, and impacts of the food matrix all affect quercetin’s bioavailability [27]. An aqueous quercetin solution administered to rats had a C_max_ of 2.01 μM and absolute bioavailability of 16%. When quercetin aglycone was administered, its C_max_ reached 3.44 μM, and its absolute bioavailability rose to 27.5% when dissolved in a solution of ethanol and PEG 200 [28]. In addition, it is widely available, effective against various diseases including cancer, and has low toxicity compared with other compounds. Thus, quercetin has been investigated as an alternative therapy in cancer treatment, offering preventive and therapeutic benefits through multiple mechanisms [29,30]. For instance, quercetin enhances apoptosis and autophagy in cancer by reducing β-catenin expression, stabilizing hypoxia-inducible factor 1 alpha (HIF-1α), inhibiting Akt, mTOR, and extracellular signal-regulated kinase (ERK) activation, and activating caspase-3 [30]. Moreover, by decreasing the secretion of MMP and VEGF. By targeting mitochondria and lowering bioenergy, it also triggers cancer cell death [31]. To inhibit apoptosis, flavonoids such as luteolin and quercetin upregulate caspase-3, p21, and Bax while inhibiting Akt, cyclin-A, cyclin-B1, Polo-like kinase-1 (PLK-1), CDC-2, cyclin-dependent kinase 2 (CDK-2), and Bcl-2 [30,32]. It decreases STAT3 activation and boosts STAT3 protein degradation in hepatic cancer cells [33].

**Figure 1:**
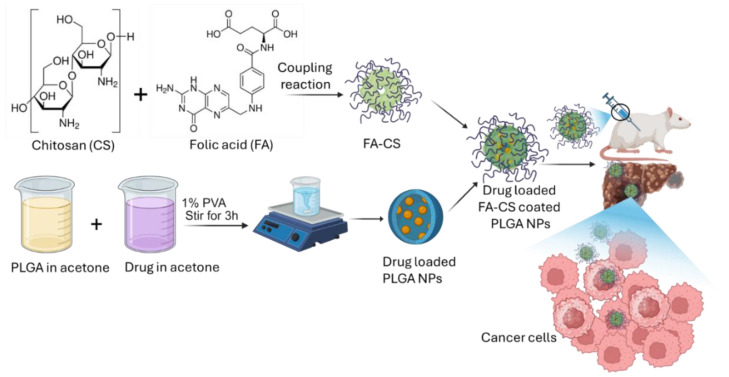
Representation of the systematic preparation of QPCF-NPs and their drug delivery for targeted hepatocellular carcinoma.

Based on the above, we hypothesized that quercetin-loaded PLGA NPs coated with CS-FA help in targeting cancer cells and also have improved drug bioavailability for sustained drug delivery for HCC treatment. This work is based on the hypothesis that modified quercetin (polylactic-co-glycolic acid) and chitosan-FA nanoparticles may work in concert to inhibit the growth of HepG2 cell lines and produce other positive effects related to hepatic carcinoma in a rat animal model (Figure 1). This hypothesis is motivated by the benefits of modified CS-FA-coated PLGA NPs loaded with quercetin and the efficacy of different molecular weights of chitosan and FA in the biomedical field. CS-FA made it possible to develop innovative systems that can target malignant regions and discharge their load there selectively while leaving the surrounding tissue unaffected. These methods make use of the pH sensitivity and folate receptors, two distinct characteristics of tumor tissues. It is anticipated that this method will increase patient compliance and adherence to treatment while also decreasing the negative effects of chemotherapy and increasing its effectiveness.

## 2. Materials and Methods

### 2.1. Chemicals and Reagents

PLGA (Mw~1000 Da; polylactic acid glycolic acid 50/50 [*w*/*w*]) was purchased from Sigma-Aldrich (St. Louis, MI, USA). Di-sodium hydrogen phosphate, potassium dihydrogen phosphate, sodium citrate, and trichloroacetic acid (TCA), FA, chitosan, EDC, NHS, PVA, sulfonic acid, and PVA were bought from SD Fine Chemicals Pvt. Ltd. located in Mumbai, India. Anhydrous methanol and polyphosphoric acid were procured from Loba Chemical Pvt. Ltd., New Delhi, India. Diethyl nitrosamine (DEN), 2,4-dinitrophenylhydrazine, and p-iodoaniline were acquired from Sigma-Aldrich, Bengaluru, India. Loba Chemicals, based in New Delhi, India, provided Tri’s buffer, sodium carbonate, 5,5-dithiotris-2-nitrobenzoic acid, glacial metaphosphoric acid, and sodium potassium tartrate. Quercetin was obtained from Sigma-Aldrich, Karnataka, India. We purchased the Aspartate Amino Transferase (AST) and Alanine Amino Transferase (ALT) kits from Trans Asia Biomedicals Pvt. Ltd. in Baddi, India. The LDH kit was procured from the LDH1169B Mod. IFCC method, and CK from the tulip group coral clinical system, Santacruz, the cox-2 kit was procured from Chongqing Biospes Co., Ltd. (Chongqing, China), and the IL-2 and IL-6 kits were purchased from Sigma-Aldrich; the caspase-3 kit was procured from Invitrogen. The supplier of the other chemicals was HiMedia in Mumbai, India. For all experiments, in-house distilled water was utilized, and all chemicals and solvents were analytical grade and 99.0% pure.

### 2.2. Preparation of Quercetin-Loaded Drug-Loaded PLGA NPs and Coating with Chitosan-FA

**Figure 2:**
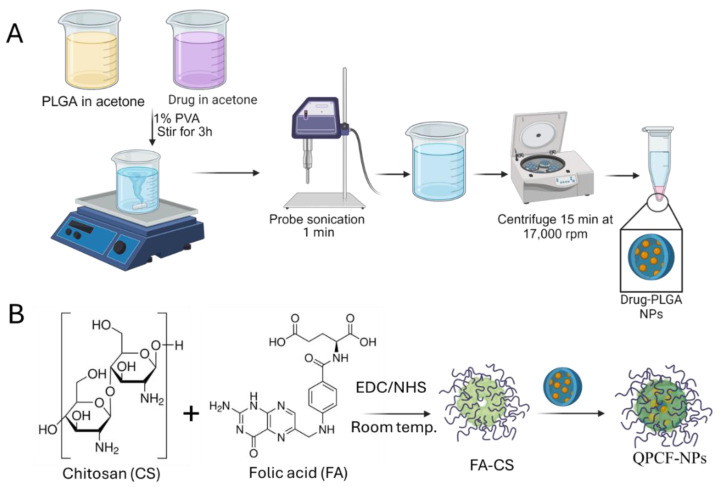
(**A**) Systematic preparation of QPCF-NPs using the emulsification evaporation method and (**B**) surface decoration by a coupling reaction.

Using an emulsion solvent evaporation method, PLGA-NPs were developed as previously described [34]. For this, 2 mL of acetone was used to dissolve PLGA and quercetin in a 2:0.1 ratio, and the mixture was then incubated for three hours at room temperature with continuous stirring. This solution was sonicated using a probe sonicator (60%, 1 min) after being introduced dropwise to a 1% PVA solution (4 mL). Additionally, the acetone was eliminated from this pre-emulsion by stirring it all night. Centrifugation was used to purify the NPs for five minutes at 4500 rpm, and the supernatant was ultracentrifuged for twenty to thirty minutes at 17,000 rpm (Figure 2A). The collected pellets were redispersed in purified distilled water to remove the unbound drug. Prior to scattering the pellets in 5 mL of pure distilled water, this process for purification was carried out 3 times. Prior to freeze-drying, the sample was stored in a freezer for 2 h at −20 °C and then 4 h at −80 °C. The resulting PLGA-NPs were freeze-dried and then kept at 4 °C for storage. Similar steps were taken to prepare blank PLGA-NPs without the inclusion of quercetin.

Folic acid (FA)-CS were conjugated by amide bond formation. Using EDC/NHS crosslinking, the -COOH groups of FA were covalently bonded to the (-NH_2_) amine end group of chitosan (Figure 2B). To conjugate, freeze-dried CS-FA were coated on the surface of drug-loaded PLGA-NPs to produce QPCF-NPs using EDC/NHS crosslinking. To activate the (-COOH) carboxyl groups of the FA, 2 mg of freeze-dried FA were dissolved in MES (0.1 M, pH 6), followed by the addition of 2 mM of EDC and 10 min of stirring. Finally, a 5 mM NHS solution was added and mixed for 20 min, followed by the addition of chitosan and another 4 h of stirring. After centrifuging at 16,000 rpm for 30 min, CS-FA were recovered and cleaned three times with PBS.

The optimized concentration of quercetin-loaded PLGA was used as a negative charge on polymeric biomaterials for surface layer coating by negative charge CS-FA. First, under continuous stirring, QP-NPs were poured dropwise into a CS-FA solution. The FA-containing solution was then mixed to synthesize the QPCF-NPs and to investigate the NPs’ activities against HCC. In the present study, QPCF-NPs were synthesized using the solvent evaporation method. This method is used for synthesizing nanoparticles with low cost and eco-friendly. The prepared NPs were examined by FTIR and XRD. Their morphology and surface architecture were examined by SEM and TEM. To track the releasing behavior of drugs from the NPs, the quercetin-releasing profile was determined. An in vitro assay in the HepG2 cell line was carried out to investigate the anti-HCC potency of QPCF-NPs.

### 2.3. Characterization of Prepared QPCF-NPs NPs

#### 2.3.1. Particle Size and Zeta Potential

A zeta sizer (Malvern paralytical) was employed to measure the zeta potential, size distribution, and particle size of PLGA-NPs and QPCF-NPs. In brief, 1.0 mg of PLGA-NPs and QPCF-NPs were re-suspended in 1 mL of distilled water. After that, these solutions were diluted at 1:50 using distilled water as a solvent. Following dilution with distilled water, all measurements were made in triplicate at 25 °C, a 90° scattering angle, a refractive index (RI) of 1.33, and a viscosity of 0.89 cp. Moreover, zeta potential estimation of NPs was carried out using a similar solution preparation. To this end, scattering was measured using a zeta sizer (Malvern Instrument, Malvern, UK). The analysis was performed in triplicate.

#### 2.3.2. FTIR and XRD Analysis of QPCF-NPs

To determine whether quercetin and the polymers interacted, FTIR measurement was performed. Separately, FTIR spectra of PLGA, chitosan, FA, PLGA NPs, and QPCF-NPs were recorded. In all, ~2 mg of compounds was pelletized at 1000 psi. Lastly, FTIR of prepared NPs was analyzed and recorded through Perkin Elmer Spectrum Two over a wave number range of 4000–400 cm^−1^. Additionally, X-ray diffraction (XRD) of the prepared PLGA, chitosan, FA, and PLGA NPs was performed, and after analyzing QPCF-NPs, the samples were retained as the surface of the copper anode material for additional examination. XRD-embedded software (DIFFRAC.EVA V7) was used to record all the data.

#### 2.3.3. SEM and HR-TEM Analysis of QPCF-NPs

SEM analyses were acquired by JSM-6490LV, JEOL, Tokyo, Japan. NPs were mounted on copper-coated grids (Ted Pella, Inc., Redding, CA, USA) and then dried for SEM. The surface morphology of the prepared QPCF-NPs was examined through SEM. The surface inner morphology and QPCF-NPs particle size were assessed using TEM at 120 kV utilizing JEOL 2011 TEM, JEOL Ltd., Tokyo, Japan. HR-TEM was used to study the surface topography and internal configuration of NPs. The size of prepared NPs within the promising range of convenient delivery. The prepared QPCF-NPs have a uniform distribution. The NPs were consistently coated with FA-CS, as evidenced by their size and smooth surface.

#### 2.3.4. HPLC Analysis

QPCF-NPs’ entrapment efficiency (EE) was evaluated by a Shimadzu LC-20AD (Kyoto, Japan) HPLC operational with a photodiode array detector. Acetonitrile (ACN): water at a 6:4 ratio with gradient elution and a flow rate of 1 mL/min comprises the mobile phase. A reversed-phase (RP)-C18 column (5.0 μm × 4.6 mm × 250 mm) and a max of 270 nm were used for the separation. Throughout the experiment, the temperature was kept at 40 °C. The overall running time was 15 min. After each run, a blank ACN injection was used to wash the column in an ACN:H_2_O = 50:50 elution solvent ratio. The supernatant was collected using a refrigerated centrifuge that was set to 4 °C and 20,000 rpm for 20 min. The quercetin mass was determined using HPLC (Figure S1) both before and after nanoparticulation (Figure S1). The EE and the actual % drug loading capacity (DL) were calculated.

#### 2.3.5. Drug Loading and Entrapment Efficiency of QPCF-NPs

Ultracentrifugation was used to determine the %EE of QPCF-NPs. The amount of unentrapped quercetin from the prepared NPs was analyzed at λ = 570 nm by UV spectroscopy after centrifuging 10 mL samples at 15,000 rpm for 15 min at 4 °C. The %EE and DL were calculated using the following formula:

(1) $$\%\;entrapment\;efficiency=\frac{Mass\;of\;drug\;originally\;taken-Mass\;of\;drug\;in\;supernatant}{Mass\;of\;drug\;originally\;taken}\times 100$$

(2) $$\%\;Drug\;loading\;capacity=\frac{MW\;of\;drug}{Weight\;of\;NPs}\times 100$$

#### 2.3.6. Drug Release Profile of QPCF-NPs

QPCF-NPs were incubated in PBS (pH 7.4) according to Zhang et al. For testing in vitro release performance of QPCF-NPs, 2 mg/mL QPCF-NPs (175 μM equivalent quercetin) were added into a sealed vial and agitated at 60 rpm for 37 °C in a shaking incubator [35]. The mixture was then diluted in pH 7.4 contained phosphate buffer. Following 0, 1, 2, 3, 6, 12, 24, and 48 h of incubation, 1 mL of PBS was taken out of the vial and changed with new PBS, then the sample was subsequently separated using centrifugation. A method for measuring the absorbance at 370 nm, HPLC (Shimadzu 1800, Tokyo, Japan) was utilized to establish the amount of quercetin released into PBS.

### 2.4. In Vitro Cytotoxicity Assay of QPCF-NPs Using MTT Assay

A HepG2 tumor cell line was subjected to in vitro MTT assay for 24 and 48 h. The American Type Culture Collection was the source of the HepG2 cells, which were cultivated in DMEM (Dulbecco’s Modified Eagle Medium) contained high glucose media (Gibco Life Technologies) supplemented with 10% FBS and 1% antibiotic P/S (Gibco Life Technologies). HepG2 cells were grown and maintained in a 5% CO_2_ incubator at a temperature of 37 °C. Three independent HepG2 cultures with 5000 cells per well were used for growing for 24 h in DMEM medium (10% FBS and 1% P/S). Later, the medium was exchanged with fresh DMEM medium as well as NPs containing medium (1% FBS and 1% P/S) and kept for 24 h incubation. Then, 10 μL of MTT dye solution (5 mg/mL in PBS pH 7.4) were added to each well following four hours of incubation at 37 °C and 5% CO_2_ with the exponentially expanding cell population; formazan crystals were plainly evident. In 150 μL of DMSO, the formazan crystals that were created by the cellular inhibition of MTT were dissolved. Once mixed in a mechanical plate mixer, the lysates’ optical density was determined at 570 nm via an ELISA reader. All experimental measurements were performed in triplicate.

Cytotoxicity was determined as:

(3) $$Cell\;viability\;\left(\%\right)\;=\frac{OD\;sample\;at\;570\;nm}{OD\;control\;at\;570\;nm}\times 100\%$$

Therefore, the reading was recorded as a % of control wells in which only the media without the treated sample was considered as the control. The cellular uptake of QPCF-NPs increased compared with quercetin, while cell viability was reduced.

### 2.5. Animal Study

The principle was previously authorized by the Institutional Animal Ethical Committee; albino Wistar rats (male) weighing 150 g–200 g were granted (Approval No. KUDOPS/182). Standard laboratory settings were maintained, with a temperature of 25 ± 5 °C and a 12 h light/dark cycle. Animals had unlimited access to a commercial pellet diet and water. The animals were kept in housing for a week before completing the experiment. Five groups of six (*n* = 6) animals were randomly selected. The following experimental groups were defined based on administration of a 42-day standard care course (SC) suspension in water: Group I received 0.25% CMC (2 mL/kg), and Group II received DEN (8 mg/kg) [36].

The protocol described herein provides an animal model designed to mimic the pathophysiological features of liver disease that leads to HCC in humans, where a combination of etiological factors, including genotoxic injury and advanced fibrosis, are likely to contribute [37,38,39]. As reported, in the DEN administered group, cyclin E, cdk, p2^CIP1/WAF1^, *p*-cdk1, and cyclin B were more highly overexpressed than in controls. DEN irreversibly induced hepatocellular carcinogenesis via overexpression of G1/S-phase regulatory proteins in rats [40]. A well-known model of liver carcinogenesis induced by a single injection of DEN, a genotoxic agent.

Group III received DEN + quercetin (50 mg/kg, subcutaneous route), Group IV received DEN + QPCFNPs (10 mg/kg, SC), and Group V received DEN + QPCF (25 mg/kg, SC). Blood was taken for additional biochemical calculations 42 days later. The liver was removed after euthanizing animals by cervical dislocation, cleaned with ice-cold saline, and preserved at 20 °C for further experiments.

### 2.6. Biochemical Analysis

A commercial kit was used to assess the amount of plasma AST and ALT [41]. As per manufacturer’s instructions, 100 μL of plasma samples were mixed with 1 mL of the working reagent, and the absorbance was measured three times at 340 nm at regular intervals of 1 min. ALT or AST activity was computed as follows:

(4) Activity of ALT or AST (U/mL) =∆A340/min×1768

A commercially available kit was used to estimate the levels of LDH and CK in plasma. LDH was procured from LDH1169B Mod. IFCC metroland CK from the tulip group coral clinical system, Santa Cruz. 100 μL of plasma samples were added to 1000 μL of working reagent in accordance with the manufacturer’s procedure, and the absorbance at 340 nm was monitored every 1 min for three minutes.

(5) Activity of LDH in (U/L) at 37 °C =∆A340/min×16030

(6) Activity of CK in (U/L) at 37 °C =∆A340/min×8095

### 2.7. Oxidative Stress Parameters in Hepatic Tissue

#### 2.7.1. Tissue GSH

In a tube, 0.2 mL of homogenate tissue, 10% (*w*/*v*) was mixed with 1.8 mL of D/W. Simultaneously, a precipitating solution was prepared by mixing 0.2 g EDTA, 30 g NaCl, and 1.67 g of glacial metaphosphoric acid in 100 mL D/W. This precipitating solution was poured into the above mixture, incubated for 5 min, and then filtered. Subsequently, 1 mL of 0.4% (*w*/*v*) DTNB and 8 mL of 0.3 M PBS were further added to 2 mL of filtrate, and the mixture was centrifuged for 1 min at 13,000 rpm. A blank was prepared without tissue samples [42].

(7) $$GSH\;(\mu M/\mu g\;of\;protein)\;=\frac{\left(310.4\times Ei\times OD\;at\;412\;nm\right)}{\mu g\;of\;protein}$$

where Ei is a correction factor around 0.542.

#### 2.7.2. Tissue PC

A 10% tissue homogenate was prepared in distilled water. A total of 150 μL of tissue homogenate was precipitated by appending 500 μL of 10% trichloroacetic acid (TCA) and centrifugation for 2 min at 13,000 rpm before discarding the supernatant. Then, cell pellets were incubated with 500 μL of 0.2% di-nitro phenyl hydrazine under regular vortexing every 5 min for 1 h. After removing the supernatant, the cell pellets were washed with 500 μL of ethanol: ethyl acetate contained (1:1) solution. Ultimately, 600 μL of guanidine HCl was used to dissolve the pellets [43], and finally, the absorbance was recorded at 360 nm.

(8) $$CalculationPC(\mu M)\;=\frac{Absorbance}{\mu g\;of\;protein}$$

#### 2.7.3. Tissue SOD

The pH = 8.5 Tris HCl buffer was mixed with 10% cytosolic supernatant (100 μL), and the ending volume was altered to 3 mL using the same buffer. After adding pyrogallol (25 μL), the absorbance change at 420 nm was measured for three minutes at 1 min intervals. A blank was created without the tissue sample [44]. The occurrence of SOD inhibits the increase in absorbance at 420 nm following the addition of pyrogallol.

(9) $$Unit\;of\;SOD/\mu g\;or\;protein\;\;=\frac{\left[100\times \{(A-B)/(A\times 50)\}\right]}{\mu g\;of\;protein}$$

where A indicates change in absorbance/min in control and B shows the change in absorbance/min in the test sample.

#### 2.7.4. Tissue CAT

The 10% tissue homogenate in 50 mM PBS was used and then centrifuged for 20 min at 10,000 rpm. Next, a cuvette including 19 mM/L, 2.95 mL of hydrogen peroxide (H_2_O_2_) solution made in potassium phosphate buffer was filled with 50 μL of the supernatant [44]. H_2_O_2_ disappearance was observed at 1 min intervals for 3 min at 240 nm. The catalase property was then calculated as:

(10) $$nM\;of\;{H_{2}O}_{2}\;disappeared/min/\mu g\;of\;protein=\frac{\left(\Delta A/min\times volume\;of\;assay\right)}{\left(0.0719\times volume\;of\;sample\times \mu g\;of\;protein\right)}$$

#### 2.7.5. Tissue MDA/TBARS

The 10% (*w*/*v*) 1 mL of tissue homogenate was mixed with 0.5 mL of 30% TCA and 0.5 mL of 0.8% Thio barbituric acid in an aluminum foil-covered tube. The tubes were allowed to incubate for 30 min at 80 °C in a shaking water bath. Then, the tubes were centrifuged for 15 min at 3000 rpm after cooling for 15 min [45]. The absorbance was measured at 540 nm alongside a blank tissue sample lacking. The MDA amount in a sample was evaluated corresponding to the following equation:

(11) $$nM\;of\;MDA/\mu g\;of\;protein\;\;=\frac{\left(V\times OD\;at\;540\;nM\right)}{\left(0.56\times protein\;concentration\right)}$$

where V indicates the final volume of the test solution.

### 2.8. Histopathological Examination of Hepatic Cancer Tissue After Treatment with QPCF-NPs

Histopathology was examined using a previously reported method with minor modifications. In brief, for primary fixation, liver tissue samples (2–4 mm) were fixed for 2–6 h in 2.5% glutaraldehyde at 4 °C. Then, samples were washed for 15 min at 4 °C in 0.1 M PBS. Following that, a post-fixation of 1% osmium tetroxide was performed for 2 h at 4 °C. After washing three times at intervals of 15 min in 0.1 M PBS, samples were kept at 4 °C. Acetone at different concentrations (30, 50, 70, 90, 95, and 100%) was then used to dehydrate these samples. Then, all specimens underwent critical point drying at 31.5 °C and 1100 pressure with air drying at room temperature. Ultimately, samples were affixed to aluminum stubs using adhesive tape. Histopathological analyses using hematoxylin and eosin staining were carried out to determine morphological alterations of the liver cells after administration of QPCF-NPs and its metabolites [46]. The tissues were stored in 10% formalin overnight and treated with 70% isopropanol the next day. The tissues were then dehydrated with 100% xylene and subjected to isopropanol at different concentrations (70, 90, and 100%). After embedding the tissue samples in beeswax, a microtome was used to prepare 5 μm slices. After staining with hematoxylin and eosin, samples were examined using a microscope at a magnification of 40×.

### 2.9. Statistical Analysis

GraphPad Prism 5.0 (San Diego, CA, USA) was employed for statistical analysis. The mean ± SD is used to express all results. One-way ANOVA (analysis of variances) and the Bonferroni multiple comparison test were employed to evaluate the data. The significant differences from the D control were taken into account for biochemical estimations (*** *p* < 0.001, ** *p* < 0.01, and * *p* < 0.05).

## 3. Results and Discussion

### 3.1. Characterization of QPCF-NPs

#### 3.1.1. Particle Size and Zeta Potential of QPCF-NPs

**Figure 3:**
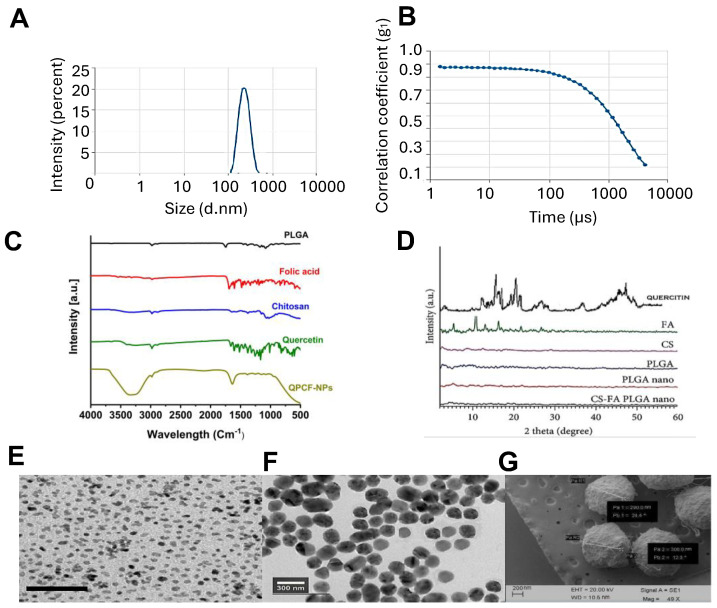
Characterization of QPCF-NPs. (**A**). Particle size of QPCF-NPs, (**B**). Correlation coefficient. (**C**). FTIR analysis of PLGA, FA, CS, QU, and QPCF-NPs. (**D**). XRD patterns of FA/CS-drug-loaded PLGA nanoparticles and (**E**,**F**). TEM QPCF-NPs. Scale bar: black = 100 nm and white = 300 nm. (**G**). SEM QPCF-NPs.

The particle size, PDI, and zeta potential determined by Malian Zetasizer software are shown in Table 1. The prepared NPs were observed in the nanoscale. Uncoated QP-NPs were smaller than after coating with CS-FA. The particle size increased from 192 ± 14.8 nm to 290 ± 0.5 nm (Figure 3A), and the zeta potential increased from −17.06 ± 1.1 mV to 24.6 ± 0.06 mV. While the coated NPs have a positive charge because of the extremely positive charge of the amine groups on the chitosan moiety and the negative charge of the -COOH end groups on the FA moiety, the uncoated NPs have a negative charge because of the -COOH end groups in the PLGA polymer. Additionally, all of the QPCF-NPs displayed minimal PDIs (Figure 3B), suggesting that the system is extremely monodispersed, as advised by nanomedicine [47]. The prepared QPCF-NPs showed the positive zeta potential (ZP) values, most perhaps associated with the free amine groups of the CS-FA-coating PLGA NPs. A similar result for PLGA NPs coated with chitosan was previously reported [48].

#### 3.1.2. Drug Loading Efficiency

The loading and encapsulation effectiveness of QPCF nanoparticles was investigated next. Compared to quercetin-PLGA particles with QPCF-NPs prepared with solvent evaporation, the drug loading and encapsulation effectiveness of QPCF-NPs were marginally higher. The method, material, and system mostly affect drug loading. In PLGA NPs, a lower MW with a -COOH end group improves concentration of -COOH groups at the surface of the particle, encouraging polar collaborations with the drug molecule. Quercetin-loaded PLGA nanoparticles were fabricated by coating chitosan and FA; their effectiveness against HCC was assessed in vitro. If the drug must adsorb on the nanocarrier’s surface, the carrier’s exterior surface area is crucial to the effectiveness of drug loading. The largest amount of medicine that can be encapsulated, concerning the amount of polymer, is 60–80%.

#### 3.1.3. FTIR and XRD

The functional group interactions between PLGA, quercetin, chitosan, and FA were investigated using FTIR and a Perkin Elmer spectrometer (India). The freeze-QPCF sample was analyzed in the 4000–400 cm^−1^ range. The spectral peak absorbance was recorded and assigned as per the instrument protocol. Figure 3C represents free PLGA showing the representative bands of polymer-CH-CH_2_-CH_3_. The C-H stretching vibrations were observed at 2983.43 cm^−1^, and the carbonyl (C=O) stretching vibration and C-O stretching or C-H bending were observed at 1746.2 cm^−1^ and 1381.67 cm^−1^ respectively. In addition, C-O stretching vibration in the ester group (-COO-) of PLGA was observed at 1175.11, and C-O bending vibrations or symmetric stretching was at 1082.84 cm^−1^. Figure 3C represents FA’s characteristic IR absorption peaks at 1689.21, 1636.77, 1603.01, and 1482 cm^−1^ that were detected owing to C=O stretching in the amide I band (from the -CONH group), C=O amide stretching of the α-COO group (carboxyl group adjacent to the amide bond), and N-H bending vibrations of the -CONH group (amide II band) and phenyl ring vibrations in folic acid, respectively. Figure 3C represents the IR spectrum of chitosan. A strong band in the region 3285.55–1025.74 cm^−1^ corresponds to N-H and O-H stretching vibrations, as well as intramolecular hydrogen bonds. The absorption bands at around 3285.55 cm^−1^ and 2980.37 cm^−1^ can be attributed to symmetric and asymmetric C-H stretching vibration, respectively. The existence of residual *N*-acetyl groups was proved by the bands at nearby 1643 cm^−1^ (C=O stretching (amide I band) and 1378 cm^−1^ C-N stretching (amide III band), respectively. We did not achieve a tiny band at 1550 cm^−1^ corresponding to the N-H bending of primary amines. This is the third band characteristic of typical *N*-acetyl groups, which possibly overlapped with other bands. The band at 1550 cm^−1^ corresponds to N-H bending of the NH_2_ while that at 1153 cm^−1^ can be recognized as asymmetric C-O-C stretching. The band at 1025 cm^−1^ corresponds to C-O stretching. These bands were previously reported in the spectra of chitosan samples [49,50,51].

Figure 3C shows the FT-IR graph of the CS-FA-PLGA (QPCF-NPs) copolymer, which confirms an interaction between the amine group of FA-CS and the free COOH group of PLGA. The peak at 3338.62 cm^−1^ denoted N-H stretching, whereas that at 1633.93 cm^−1^ corresponded to the occurrence of the C=O group of the amide. The peaks at 2935, 2848.4, and 2669.5 cm^−1^ could be recognized as the occurrence of CH stretching, and the peak at 1578.6 cm^−1^ is the N-H bend. Nonetheless, the peaks at 1249.5 cm^−1^ and 1087.8 cm^−1^ indicated the presence of C-O in chitosan and PLGA [52]. The quercetin infrared spectrum in Figure 3C shows its separate components. A peak at 3249 cm^−1^ corresponds to stretching absorption from the related hydroxyl group in pure quercetin, while 1606 cm^−1^ was recognized as C=O stretching, 1257 cm^−1^ as C-H stretching, and 1166 cm^−1^ as C-O-C stretching. The peaks at 1633 cm^−1^ indicate C=O absorption, and the 1618 cm^−1^ band indicates C-C stretching, while the peak at 1244 cm^−1^ can be recognized as stretching (-C-O) of the oxygen in the ring. Similarly, -C-OH- twist vibrations were observed at 1317 cm^−1^, and the peaks at 1163–1002 cm^−1^ account for C-O stretching. These observations are similar to previously published spectra [53].

The free PLGA NPs spectra revealed the typical bands of the polymer: -CH, and -CH_2_, -CH_3_ stretching, which were monitored in the 3300–2900 cm^−1^ range, and carbonyl-C=O and C-O stretching in the 1600–1300 cm^−1^ and 1200–1100 cm^−1^ ranges. In addition, bending stretching for -CH_3_ was noted around 1633–1381 cm^−1^. OH groups (3300–2900 cm^−1^), C=O absorption (1381 cm^−1^), C=C aromatic stretching bands (1633–1381 cm^−1^), C-H bending in the plane at 1050–950 cm^−1^) and out of plane at 850–600 cm^−1^, a band ascribed to the C–O stretching of oxygen (1381–1251 cm^−1^), and the region for C-O aromatic stretching (1251–1151 cm^−1^) were all prominent features of quercetin spectra (Figure 3F) [54].These findings indicate that quercetin forms hydrogen bonds with the PLGA polymer. Furthermore, the band relating to C=O stretching (1600–1300 cm^−1^) was wider in the quercetin-loaded PLGA NP, suggesting that quercetin and the PLGA polymer are linked by interactions between the carbonyl and COO groups of quercetin and polymer. In other words, IR verified that quercetin was encapsulated in PLGA NPs.

According to XRD, quercetin and FA were found in the crystallization phase (Figure 3D), while other chemicals were in an amorphous state. The quercetin distributed into PLGA cores did not precipitate during trials. Conversely, FA was distributed at nanoscales or smaller than the nanoparticles in the outer layers of the chitosan. Quercetin and FA’s diffractogram showed several reflections, suggesting that the drug is crystalline. Quercetin reflections were observed at diffraction angles of 2 Theta (degree) at 7.5, 9.0, 11.0, and 12.2 (>1000 Cps) and in the 2–60° range. FA was thus found in intervals denoted by 10.8, 5.2, 13.1, 16.2, and 22.8. Conversely, PLGA and chitosan are colloidal materials that display amorphous polymer patterns. Quercetin and FA’s unique, crisp reflections vanished from the XRD patterns of PLGA-NPs loaded with FA/CS (Figure 3E). Thus, the drug was incorporated inside the core of QPCF-NPs.

#### 3.1.4. SEM and TEM of QPCF-NPs

The surface of the final prepared conjugate particles was examined by SEM. An irregular shape with different particle sizes of approximately 200–300 nm was observed (Figure 3E–G). The inner morphology of the particles was investigated using HR-TEM. As shown in Figure 3G, the particle was similar to the SEM image in shape and size in the TEM analysis with a smooth surface without gains. The electron point image also suggested that the particle had an amorphous-like nature (Figure 3E). Hence, according to the morphology and nature of the NPs, the prepared conjugate could be used for further drug delivery.

### 3.2. Drug Release

**Figure 4:**
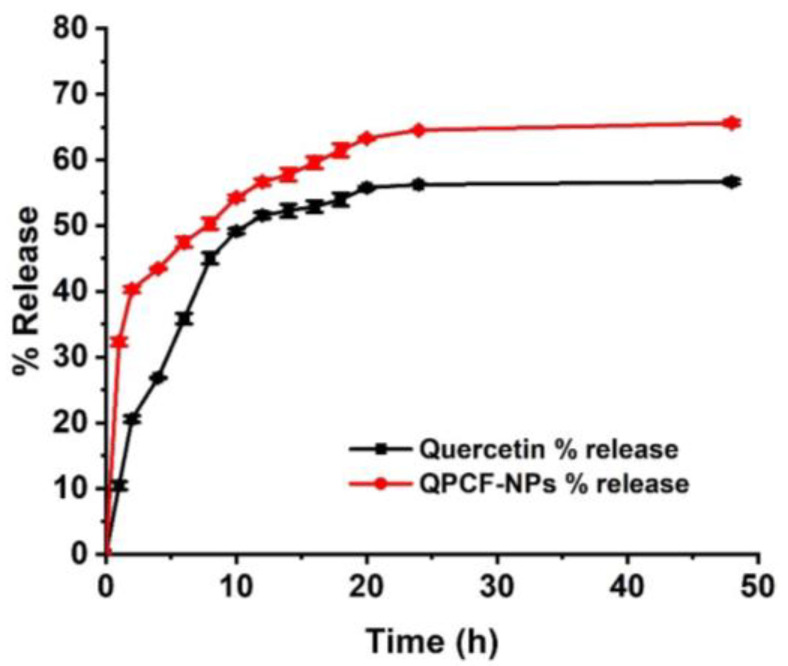
Graph showing the drug release profiles of quercetin and QPCF-NPs at pH 7.4 phosphate buffer.

Figure 4 shows the in vitro drug release profiles of QPCF-NPs. The release of QPCF-NPs increased significantly (~70%) compared to plain quercetin at 7.4 pH over 48 h. The sustained release pattern of quercetin from QPCF-NPs shows that the dissolution profile of quercetin inside the hydrophilic solvent was enhanced by biopolymer coating.

### 3.3. Cytotoxicity and Cell Apoptosis Assay

#### Cell Viability Assay

**Figure 5:**
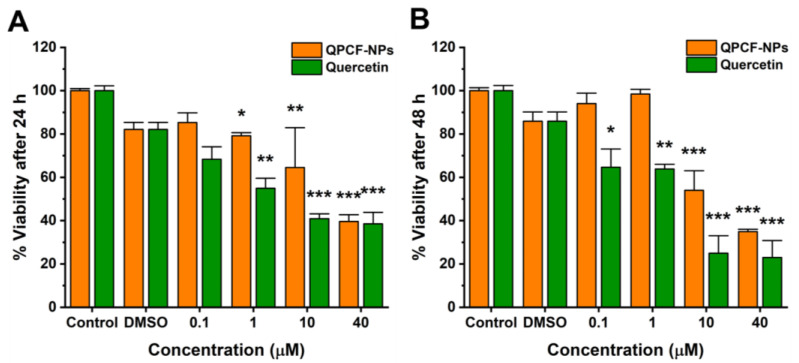
(**A**) In vitro MTT cytotoxicity assay of QPCF-NPs and quercetin in 24 and (**B**) 48 h. The standard deviation is displayed by error bars (* *p* < 0.05, ** *p* < 0.01, *** *p* < 0.001 vs. untreated controls).

A cell viability assay was carried out to investigate the effect of the prepared QPCF-NPs on the HepG2 cell line. As shown in Figure 5, After 48 h of incubation, quercetin did not reveal any appreciable variations between the treatment and control groups. QPCF-NPs showed significant differences from the control group at different concentrations. In addition, previously reported PLGA NPs did not significantly influence the viability of various cancer cell lines. Thus, PLGA-containing NPs were safe in cell experiments. Using reference medication, QPCF-NPs were evaluated against HepG2 cells. PLGA and PVA exhibited no significant in vitro cytotoxicity against HepG2 cells. Nevertheless, QPCF-NPs showed more cytotoxicity than quercetin alone.

### 3.4. Determination of Antioxidative Effects of QPCF-NPs

**Figure 6:**
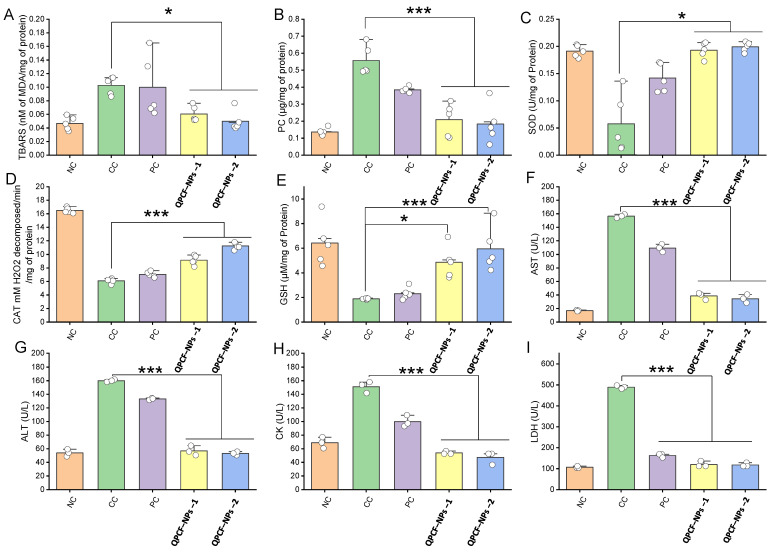
(**A**–**I**) Effect of QPCF-NPs (SC, 10 and 25 mg kg^−1^ for 15 days) on TBARS, PC, SOD, CAT, and GSH in liver tissue (data is shown as mean ± SD (*n* = 6)) and AST, ALT, CK, and LDH plasma level induced by DEN using it as a carcinogen. (**E**–**H**) impact of QPCF-NPs on plasma enzyme levels following dosages of 10 and 25 mg/kg over a 15-day period. The data is shown as mean ± SD (*n* = 3). Statistically significant variations were identified between carcinogen control-positive and test groups [one-way ANOVA followed by Bonferroni multiple comparison test (* *p* < 0.05, *** *p* < 0.001)].

To test various malignant states for differential diagnosis, prognosis, and the advancement of cancer therapy, various antioxidant parameters—glutathione, malondialdehyde, catalase, superoxide dismutase, and protein carbonyl (GSH, MDA, CAT, SOD, and PC)—were tested to assess malignant states [42,43,44,45] using two different doses of QPCF-Nps-1 (10 mg/kg) and QPCF-NPs-2 (25 mg/kg). Tissue MDA levels were measured to demonstrate the protective effect of QPCF-NPs. Lipid oxidation is a crucial metric for assessing oxidative stress-induced liver damage. According to our findings, the tissue MDA level was higher in rats receiving DEN injections, decreasing in rats receiving QPCF-NPs (Figure 6A). Then, a PC experiment was conducted to investigate the association between the protective effect of QPCF-NPs and oxidative stress during HCC. The DEN-treated group created a greater amount of PC. Reactive oxygen species production oxidizes the carboxyl group of proteins, converting it to PC, a crucial marker for oxidative stress-induced damage in malignant cells. PC formation was significantly reduced in rats treated with QPCF-NPs (Figure 6B). To assess the preventive effect of QPCF-NPs, we measured SOD levels in the liver. Superoxide free radicals are neutralized in normal physiological conditions by SOD, an enzyme that scavenges free radicals. SOD levels were reduced in rats treated with DEN, but in those treated with QPCF-NPs, the levels normalized (Figure 6C). Next, we measured SOD and CAT liver levels. An essential oxidative enzyme found in the liver, CAT catalyzes the transformation of H_2_O_2_ into equivalent amounts of oxygen and water. Peroxides and reactive oxygen species cause this enzyme’s activity to decrease. The increase in H_2_O_2_ in rats receiving QPCF-NPs demonstrated that the liver had more CAT enzyme available to break down H_2_O_2_. When compared to free quercetin, quercetin NPs had a stronger anti-tumor activity in tumor-bearing animals. When compared to free quercetin, the IC_50_ value of quercetin-loaded chitosan nanoparticles was considerably lower in the in vitro cytotoxicity assay [55]. Superoxide dismutase (SOD) levels in the serum of tumor-bearing mice treated with quercetin-loaded chitosan nanoparticles were significantly higher than those of the free quercetin-treated group [56]. This experiment indirectly suggested that QPCF-NP treatment can increase the liver CAT enzyme levels (Figure 6D).

According to McIntyre and Rosalki [57], there is a strong link between altered cells and malignant conditions in these parameters. Therefore, we performed a variety of biochemical assessments of the liver to assess the toxicity mechanism and determine the preventive effect of QPCF-NPs against hepatic cancer. More GSH was depleted in DEN-treated rats than in the control group (NC) (Figure 6E). The administered QPCF-NPs at 10 and 25 mg/kg in rats returned their GSH levels to normal. The tripeptide GSH is more prevalent in the liver and other tissues. In the oxidation-reduction process, GSH plays a key role in scavenging free radicals during oxidative damage [57]. This result suggests that QPCF-NPs may be useful in preventing the liver damage caused by DEN. Antioxidant parameters such as GSH, PC, SOD, TBARS, and CAT are some of the oxidative stress markers measured in the liver tissue to assess the anti-proliferative potential of QPCF-NPs. GSH levels in toxic control (~3.40 μM) were significantly lower than those in the normal control (~6.4 μM). Following the administration of QPCF-NP, there was an improvement in the GSH level (~4.85 μM for 10 mg/kg and ~5.9 μM for 25 mg/kg). A similar pattern was recorded for SOD, where the toxic control group’s (CC) SOD level dropped to 20–30% compared to the normal group, while QPCF-NPs treatment increased this level to 80–90%. Similar findings were found for the CAT assay, demonstrating increased enzyme activity for both doses in the nanoformulation-treated groups compared to the toxic control (CC) and positive control groups. Separately, we measured tissue. To assess QPCF-NPs’ protective effect, MDA and PC formation are used. For toxic control, MDA formation was approximately 0.10 nM; however, when QPCF-NPs were administered, this formation decreased to approximately 0.6 and 0.5 nM. Once more, we found that PC production was greater for the toxic control (~0.55 μM) and decreased by half for the rats treated with QPCF-NPs (~0.20 and 0.18 μM) of 3.5.

Plasma AST, ALT, LDH, and CK levels increased almost twofold in CC rates compared to quercetin (Figure 6). These effects were found to decrease as previously reported, indicating the potency of quercetin to regulate the biochemical parameters [58]. Following SC administration with QPCF-NPs at 10 and 25 mg/kg, respectively, the plasma level of these enzymes was considerably (*p* < 0.001) reduced in comparison to CC rats. We measured plasma AST, ALT, LDH, and CK levels to assess the antiproliferative effect of QPCF-NPs. Our findings indicated that rats treated with QPCF-NPs had lower enzyme levels than the harmful control group. The two main liver enzymes removed during liver injury are AST and ALT [59]. DEN injection in rats increased these enzyme levels in their plasma, indicating liver injury (Figure 6F–I), while QPCF-NPs demonstrated protective activity and enzyme levels fell concurrently.

### 3.5. ELISA of COX-2, IL-2, IL-6, and Caspase-3

All inflammatory cytokines increased in DEN-exposed rats and decreased to a specific level after QPCF-NP treatment. Unlike IL-2, IL-6, and COX-2, the level in DEN, an exposed group, was suddenly raised and more quickly returned to normal after receiving QPCF-NPs. This showed that the development of HCC is significantly influenced by the expression of inflammatory cytokines; our recently identified QPCF-NPs can primarily function by reducing cytokine expression at tumor locations.

**Figure 7:**
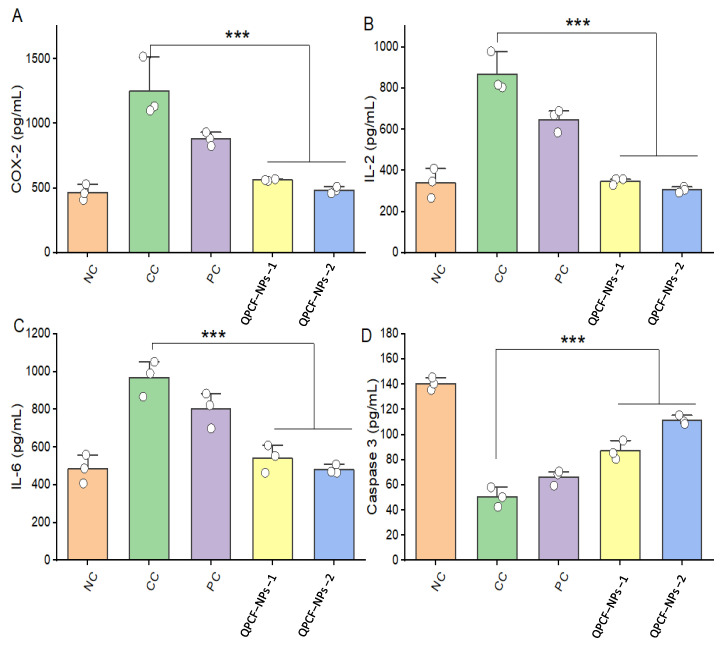
Pro-/anti-inflammatory cytokine levels (**A**) COX-2, (**B**) IL-2, (**C**) IL-6 & anti-apoptotic protein levels (**D**) Casepase-3. Data from the ELISA experiment are shown as mean ± SD (*n* = 6). There were statistically significant variations between the test and carcinogen control-positive groups [one-way ANOVA followed by Bonferroni multiple comparison test (*** *p* < 0.001)].

After measuring pro-/anti-inflammatory cytokine levels in rats’ serum, all three pro-inflammatory cytokines (IL-2, IL-6, and COX-2) were markedly elevated by DEN [60]. Apoptotic markers (caspase-3) were compared with the quercetin group and significantly suppressed the secretion of anti-inflammatory cytokines. QPCF-NPs could significantly decrease IL-2, IL-6, and COX-2 serum concentrations while significantly increasing the level compared with the DEN group (Figure 7). This indicated that hepatic cancer resulted from decreased caspase-3 expression due to downstream regulation of protein expression and upstream regulation of anti-apoptotic protein levels. Quercetin’s anti-cancer properties include promoting apoptosis, autophagy, and loss of cell viability by modulating the PI3K/Akt/mTOR, Wnt/β-catenin, and MAPK/ERK1/2 pathways [61]. Because it can precisely target molecular pathways involved in glucose metabolism and mitochondrial function, quercetin plays a critical role in cancer metabolism [30,62]. Through a number of methods, including G1 phase arrest, tyrosine kinase inhibition, downregulation of mutant p53 proteins, and downregulation of proliferative, anti-apoptotic, and cell survival proteins, quercetin can slow the growth of cancer [30,31]. Thus, the activation of caspase-3-mediated apoptotic signals resulting from QPCF-NP may account for its potential anti-HCC effects.

### 3.6. Histopathology and SEM

**Figure 8:**
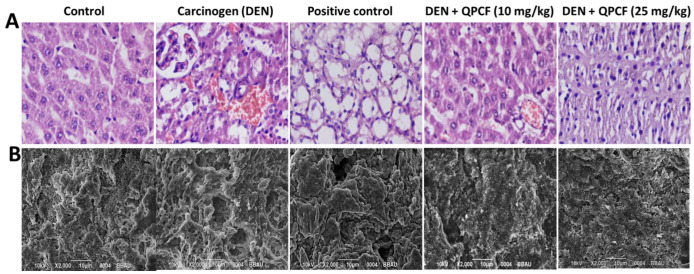
(**A**) Histopathology (**B**) Tissue SEM Analysis. The hepatic pathological alterations in rats with DEN (scale bar 50 μm). In the DEN group, tumor vacuoles were noticeable, but these disappeared following the administration of quercetin and QPCF.

Histological alterations in the hepatic tissue in the normal and numerous treatment groups were investigated using hematoxylin and eosin staining [63]. Control slices displayed the typical cell architecture and presence of nuclei. Conversely, mice exposed to DEN had livers with loss of architecture together with larger, polygonal, binucleate hepatocytes. Tumor anaplastic cells ruptured hepatic cells (RC) of Kupffer cells (KC), irregular sinusoids, a damaged nucleus, and tumoral vacuoles were specifically observed in the affected animal liver sections. Rats exposed to QPCF-NPs showed a significant improvement in the overall microscopic appearance of their hepatic tissue (Figure 8A). Additional proof of the protective effect was determined using SEM analysis and histology. Accordingly, animals treated with DEN had irregularly shaped nuclei and irregular cytoplasm. This finding suggests that DEN administration may cause excessive free radical generation [64]. Quercetin modulates various dysregulated signaling pathways, including those linked to autophagy and apoptosis, in order to achieve its anticancer effects. In particular, quercetin inhibits many signaling pathways, including PI3K/AKT, NF-κB, P53, Wnt/β-catenin, MAPK, JAK/STAT, and the Hedgehog pathway, to provide its anti-cancer actions. Quercetin disrupts many intracellular signaling molecules, including VEGF, TNF-α, Bax, Bcl-2, and caspases [65]. Breast cancer, prostate cancer, ovarian cancer, lung cancer, colon cancer, hepatocellular carcinoma, lymphoma, and pancreatic cancer are among the cancer types for which quercetin’s anticancer properties have been investigated [65,66]. It has been demonstrated that quercetin and quercetin NPs target transcriptional factors, enzymes, and receptors that are essential to the development, spread, and progression of cancer. Furthermore, it is quite interesting to see how they maintain the effects of proven chemotherapeutic drugs [65]. In comparison to the toxic control, QPCF-NP-treated animals showed fewer RC and DC, indicating a protective effect against HCC. SEM analysis revealed similar tendencies (Figure 8B).

## 4. Conclusions

Here, we determined the molecular mechanism behind the anticancer efficacy of our recently announced drug, modified effectively employed a strategic blending of biopolymers. This research determined the antitumoral mechanism of action of QPCF-NPs. The newly published in vitro study is supported by our in vivo outcome. The anticancer potential of QPCF-NPs through suppression of IL-2 and IL-6-mediated phosphorylation/activation of Caspase-3 was significantly validated by ELISA. In vivo and in vitro studies, which exhibit tumor suppression and inhibition results. Also validate the efficacy of our drug modification strategy. Furthermore, the lack of toxicity in mice increases drug bioavailability. QPCF-NPs were successfully prepared for targeting hepatocellular carcinoma. Our results indicated that QPCF-NPs improved the antioxidant state and normalized several pathological enzymes. Morphological-histopathological studies support that QPCF-NPs scavenged free radicals generated through DEN and maintained normal tissue architecture. Again, the antiproliferative action might be due to binding to caspase-3. HPLC analyses revealed that QPCF-NPs had good absorption and liver tissue distribution, indicating the initiation of apoptosis in HCC. It was also observed that QPCF-NPs deposited in the liver but showed their protective action against HCC. Therefore, QPCF-NPs might be effective against DEN-induced liver carcinoma in rats, which is beneficial for future drug design. In conclusion, our analysis emphasizes the importance of carefully planning formulations and drug polymer modification’s effective potential to treat hepatic cancer. This opens the door for further investigation into these effective therapy approaches, which could lead to the creation of more potent and practically feasible QPCF-NPs and possibly other difficult cancer treatments. The purpose of this study is to create and assess folic acid-chitosan-coated PLGA nanoparticles loaded with quercetin in order to improve drug delivery and targeting in the treatment of HCC. With the help of chitosan’s biocompatibility and folic acid’s targeting ability, this innovative nanoparticle formulation aims to reduce adverse effects and increase therapeutic efficacy. The findings show notable enhancements in drug release kinetics, cellular uptake, and anticancer effectiveness in HCC cells, indicating the possibility of this tailored nanotherapy for more successful HCC treatment.

## Figures and Tables

**Table 1:** Average diameter, average polydispersity index (PdI), and ZP of PLGA-NPs and QPCF-NPs.

NPS	Probe Sonication Times (s)	Drug:Polymer Ratio	Average Diameter (nm)	Average PdI (nm)	Zeta Potential (mV)
PLGA-NPS	60 s	0:5	192 ± 14	0.2001 ± 0.171	−21 ± 15
QPCF-NPs-1	60 s	1:5	290 ± 0.055	0.039 ± 0.016	36 ± 0.43

## Data Availability

Data are contained within the article.

## Supplementary Materials

The following supporting information can be downloaded at: https://www.mdpi.com/article/10.3390/polym17070955/s1, Figure S1: HPLC analysis of A. Quercetin B. QPCF-NPs and QPCF retention Time.

## Abbreviations

The following abbreviations are used in this manuscript:

HCC	Hepatocellular carcinoma
FA	Folic acid
PLGA	Polylactic-co-glycolic acid
RP	Reversed-phase
EE	Entrapment efficiency
QPCF-NPs	Quercetin PLGA-coated folic acid nanoparticles
FTIR	Fourier-Transform infrared
SEM	Scanning electron microscopy
TEM	Transmission electron microscopy
XRD	X-ray diffraction
MDM2	Mouse double minute-2 homolog
PARP	Poly (adenosine diphosphate-ribose) polymerase
HIF-1α	Hypoxia-inducible factor 1 alpha
ERK	Extracellular signal-regulated kinase
CDK-2	Cyclin-dependent kinase 2
PLK-1	Polo-like kinase-1
TCA	Trichloroacetic acid
DEN	Diethyl nitrosamine
AST	Aspartate Amino Transferase
ALT	Alanine Amino Transferase
GSH	Glutathione
MDA	Malondialdehyde
CAT	Catalase
SOD	Superoxide dismutase
PC	Protein carbonyl
