# Supplementary material for: Chitosan-Folic Acid-Coated Quercetin-Loaded PLGA Nanoparticles for Hepatic Carcinoma Treatment
Source: Polymers (Basel). 2025 Mar 31;17(7):955. doi: 10.3390/polym17070955 (PMC11991491; doi:10.3390/polym17070955)
Supplement: Supplementary file 1 [file polymers-17-00955-s001.zip › polymers-3561356-supplementary.pdf]

Supplementary data

Chitosan-Folic Acid-Coated Quercetin-Loaded PLGA Nanoparticles for Hepatic Carcinoma Treatment

Anil Kumar Sahdev [1], Chaitany Jayprakash Raorane [2,*], Mohammad Ajmal Ali [3], Khalid Mashay Al-Anazi [4], Ranjith Kumar Manoharan [5], Vinit Raj [2] and Anita Singh [1,*]

[1] Department of Pharmaceutical Sciences, Faculty of Technology, Sir J.C. Bose Technical Campus, Kumaun University, Nainital 263136, Uttarakhand, India; anilsahdev20@gmail.com
[2] School of Chemical Engineering, Yeungnam University, Gyeongsan 38541, Republic of Korea; drvinitraj@cau.ac.kr
[3] Department of Botany and Microbiology, College of Science, King Saud University, Riyadh 11451, Saudi Arabia; alimohammad@ksu.edu.sa
[4] Department of Zoology, College of Science, King Saud University, Riyadh 11451, Saudi Arabia; alanazik2020@gmail.com
[5] Department of Civil Engineering, Yeungnam University, Gyeongsan 38541, Republic of Korea; mrkumarbiotech@gmail.com
* Correspondence: chaitanyaraorane22@ynu.ac.kr (C.J.R.); dranitaku@gmail.com (A.S.)

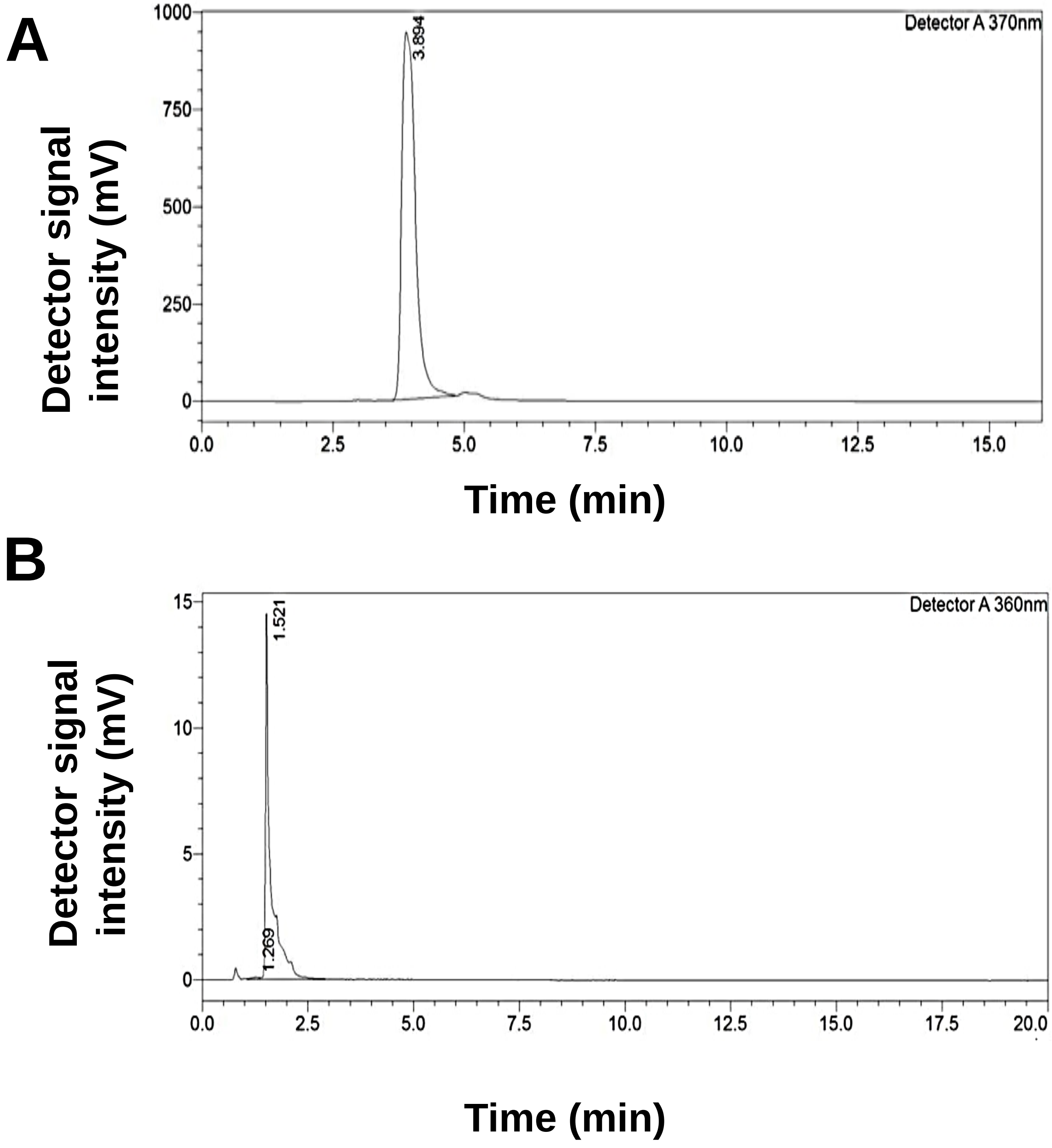

Figure S1. HPLC analysis of A. Quercetin B. QPCF-NPs and QPCF retention Time.
